# The real option value of multistage agricultural research for development

**DOI:** 10.1371/journal.pone.0336819

**Published:** 2026-09-17

**Authors:** Benjamin Schiek

**Affiliations:** Policy, Inclusion, and Socioeconomic Analysis for Impact and Learning, Alliance of Bioversity International and CIAT, Palmira, Valle del Cauca, Colombia; Public Library of Science, UNITED KINGDOM OF GREAT BRITAIN AND NORTHERN IRELAND

## Abstract

Agricultural research for development (AR4D) donors face increasingly austere fiscal outlooks that restrict their ability to commit to high impact, high risk, agricultural research projects with long time horizons. This results in a series of short-term, piecemeal funding arrangements that inhibit the AR4D community’s ability to conduct the disruptive research necessary to meet the Sustainable Development Goals. Real option valuation (ROV) presents a potential mechanism offering donors the flexibility they require to justify long term research funding commitments. However, adaptation of existing ROV methods to the AR4D context is complicated by the financial and corporate underpinnings of these methods, which are inconsistent with the non-market character of most AR4D projects. Adaptation is also complicated by the multistage structure of most AR4D projects, since existing ROV methods are mostly limited to single-stage projects. Here I present a multistage ROV method derived from novel theoretical underpinnings that are consistent with the AR4D context. As an illustrative example, I apply the derived model to evaluate a real, four-stage potato research project.

## 1. Introduction

Agricultural research for development (AR4D) projects tend to be high reward, but also high risk, propositions with long time horizons. Donors are attracted by the high potential payoff of such projects, but face increasingly austere fiscal outlooks that restrict their ability to engage in long term, risky commitments. For researchers, the resulting uncertainty in the long term funding outlook incentivizes work in the direction of low risk, modest objectives that are achievable in the near term. Such research has its place; but the Sustainable Development Goals (SDGs) call for higher impact, riskier, longer term research as well. Real options valuation (ROV), a method of project appraisal adapted from financial analysis, presents a potential enabling mechanism by which to secure long term funding for disruptive research while also guaranteeing donors the fiscal flexibility they require to justify such commitments.

The notion of a real option, first introduced by Myers [1], is born of the analogous concept of a type of financial option known as the European call option. The holder of a European call option on an underlying security (a stock, for example) has the right, but not the obligation, to purchase the security for a predetermined price—called the “strike” or “exercise” price—at the end of a certain time period. Analogously, donors who “buy” a real option on an underlying project commit to funding the project for a certain time period, at the end of which they have the right, but not the obligation, to continue funding the subsequent stage of the project. In the case of research projects, for example, the subsequent stage at the end of time period *T*_0_ might be the release and outscaling of a new crop variety developed during the main research stage. The value of such a real option (*ROV*_1_), as evaluated at the time of the investment decision (*t* = 0), may be expressed mathematically as follows.


$$\begin{array}{c} ROV_{1}(t){|}_{t=0}=e^{-rT_{0}}E[\max(x(T_{0})-K_{0},0)]{|}_{t=0} \end{array}$$ (1)

Where *x*(*T*_0_) is project net present value (NPV) at the end of the research period *T*_0_, *K*_0_ is the cost of funding the subsequent project stage (e.g., release and outscaling of the finished research product), and *r* is the discount rate. By contrast, the comparable conventional appraisal of the project is calculated as


$$\begin{array}{c} e^{-rT_{0}}(E[x(T_{0})]{|}_{t=0}-K_{0}) \end{array}$$ (2)

To characterize this as the conventional “NPV approach”, as is sometimes done in the ROV literature (e.g.[2],), can be confusing since *x*(*t*) is already defined as project NPV at any time *t* (net of *K*_0_), and appears in the calculation of ROV. To be clear, then, in the present article, the conventional manner of project appraisal in Eq (2) is referred to as the “Cost Benefit Analysis (CBA) approach” or “CBA basis”; whereas “project NPV”, denoted *x*(*t*), refers to the expected value at time *t* of the net benefit stream resulting from release and outscaling of the research product after time *T*_0_, net of any costs incurred at or prior to time *T*_0_. As in other theoretical treatments of ROV [3], the present work does not address the complexities involved in the evaluation of *x*(*t*) itself, which in the AR4D context include, e.g., non-market valuation of public goods, payment for ecosystem services, public health outcomes, nutritional security, and other intangibles (see Freeman et al. [4] for a comprehensive introduction). For present purposes, it is assumed that an evaluation of *x*(*t*) exists, typically as the result of an ex-ante impact assessment.

On an ROV basis, the investment *K*_1_ required to implement the research is justified if


$$\begin{array}{c} e^{-rT_{0}}E[\max(x(T_{0})-K_{0},0)]{|}_{t=0}>K_{1} \end{array}$$ (3)

On the CBA basis, the criterion is


$$\begin{array}{c} e^{-rT_{0}}(E[x(T_{0})]{|}_{t=0}-K_{0})>K_{1}\;\;\to \;\;\text{invest in project} \end{array}$$ (4)

ROV is typically higher than the value $e^{-rT_{0}}(E[x(T_{0})]{|}_{t=0}-K_{0})$, such that a project that would normally be rejected on the conventional CBA basis could be accepted on an ROV basis. This is especially true in the case of high risk, high reward projects. A comprehensive introduction to ROV and the main developments since its inception is given by Trigeorgis [5] and Trigeorgis and Tsekrekos [6]. For an introduction to ROV in R&D contexts, see Doctor et al. [7], and Newton et al. [8]. The agricultural ROV literature focuses on diverse aspects of farm or firm level investment options and technology adoption [2,9–12], but has not yet considered the ROV of multistage research from the perspective of donors and institutions.

Real options contracts potentially bridge the divide between donor risk aversion and the risk tolerance required to achieve high impact AR4D because they are mutually beneficial for both donors and researchers alike. From the donor’s perspective, a real option contract is a way to tentatively lay claim to promising, but distant and uncertain, research results, while at the same time guaranteeing easy extraction from underperforming projects. The resulting fiscal agility then translates into longer term funding commitments, which is music to the ears of the research community.

It is worth noting, moreover, that many donors already acquire an unacknowledged option value in their conventional research funding arrangements. That is to say, nowadays, most AR4D contracts are signed for no more than 1–5 years, at the end of which the contract comes up for renewal or cancellation, pending progress up to that point. By reserving the right to discontinue funding at specific time intervals, the donor effectively acquires option value. The introduction of ROV would thus merely formalize an already existing practice, in such cases, and more accurately appraise the real value acquired by the donor in exchange for their investment. By the same token, the default CBA methods currently in place neglect to price in the donor’s option value, and thus understate the real value acquired by the donor.

ROV methods have so far developed primarily in the context of corporate projects. A simple extension of this work to the AR4D context is complicated, if not made impossible, by the fundamental difference between the two contexts: corporate projects unfold in market or near-market settings, where much of the original option value theory developed in finance may be invoked. AR4D projects, on the other hand, typically unfold in the midst of market failures, where the underlying assumptions of such theory do not apply. In particular,

1. AR4D project NPV is not well approximated by a linear combination of price movements (the “replicating portfolios” assumption)
2. AR4D project risk cannot be hedged away by trading securities (the “complete markets” assumption)
3. AR4D donors are not willing to take on any level of risk for a given expected return (“risk neutral valuation”).

Extension of existing ROV methods to the AR4D context is also complicated by the multistage nature of most AR4D projects. For example, many AR4D plant breeding projects involve a preliminary greenhouse and/or minimal field trial stage, followed by larger multi-location, multi-season field trials [13]. In transgenic projects, additional toxicological testing is required, followed by the compilation of a regulatory dossier and application for deregulation before the national competent authority in the target countries where the new technology is to be released [14]. Finally, there is the release, distribution, and outscaling stage, which involves careful orchestration of complex networks of stakeholders and public-private partnerships.

Donors invest in each of these stages one at a time, consecutively, with investment in the subsequent stage contingent upon successful completion of the current stage. The donor’s investment in stage 1 of a 3-stage project, then, is analogous to purchasing an option on stage 2—i.e., the right but not the obligation to invest in stage 2—which is in turn an option on stage 3. The ROV of stage 1 of such a project is the value of the option to invest in stage 2. This may be expressed as follows.


$$\begin{array}{c} ROV_{3}(t){|}_{t=0}=e^{-rT_{2}}E[\max(ROV_{2}(T_{2})-K_{2},0)]{|}_{t=0} \end{array}$$ (5)

Where $ROV_{2}(T_{2})$ is the ROV of stage 2 evaluated at *T*_2_ (the end of stage 1), *K*_2_ is the cost of investing in stage 2 upon successful completion of stage 1; and stage 1 is considered successfully completed if $ROV_{2}(T_{2})>K_{2}$. And $ROV_{2}(T_{2})$, in turn, is defined


$$\begin{array}{c} ROV_{2}(T_{2})=e^{-rT_{1}}E[\max(ROV_{1}(T_{1})-K_{1},0)]{|}_{t=T_{2}} \end{array}$$ (6)

Where $ROV_{1}(T_{1})$ is the ROV of the third and last stage of research evaluated at *T*_1_ (the end of stage 2), *K*_1_ is the cost of investing in stage 3 upon successful completion of stage 2; and stage 2 is considered successfully completed if $ROV_{1}(T_{1})>K_{1}$. And $ROV_{1}(T_{1})$, in turn, is the ROV of the last stage—the value of the option to invest in release/outscaling/marketing of the finished research product with NPV *x*(*T*_0_)—as defined in Eq (1).

If *ROV*_3_(0) is greater than the investment *K*_3_ required to implement stage 1, then investment in stage 1 is justified. The comparable CBA criterion for any *n*-stage research project would be


$$\begin{array}{c} e^{-rT_{0}}E[x(T_{0})]{|}_{t=0}-\sum_{i=0}^{n-1}e^{-rT_{i}}K_{i}>K_{n}\;\;\to \;\;\text{invest in project} \end{array}$$ (7)

Where *n* = 3 in this particular example.

Existing ROV methods, meanwhile, focus primarily on single-stage options. A niche of two-stage studies (e.g.[12],) adapt the “compound option” model pioneered by Geske [15] in financial markets; and Cassimon et al. [16] extend this framework to multistage research investments (i.e., investments of arbitrary stage number) for the valuation of pharmaceutical trials. Again, such adaptations are not readily extensible to the AR4D context due to their deep roots in financial theory. A separate strand of the literature is multistage in the sense that it assesses the optimal timing of investments across multiple consecutive decision points (e.g.[17,18],). This is an adaptation of a different kind of financial call option known as the American call option, which may be exercised at any moment up to a given time limit [19]. Such methods are inapplicable for present purposes due both to their financial theoretical underpinnings and because time horizons between investment decisions in the AR4D setting are generally fixed. Breaking from the financial lineage entirely, Belz et al. [20] introduce a novel mixed-integer programming approach that accommodates multiobjective, non-financial benefit streams for the assessment of portfolios of public and private R&D projects. While this presents an interesting alternative for portfolio level assessment, the present work is concerned with ROV at the level of individual projects. Furthermore, it is concerned with research investments of arbitrary stage number, whereas Belz et al.’s discrete formulation incurs major computational challenges beyond the two-stage case.

To redress this lacuna, here I derive a method for evaluating the ROV of multistage projects in a non-market setting (e.g., Eq (5)), with particular reference to AR4D as the motivating applied context. Rather than build upon the existing ROV groundwork cited above, which is largely inherited from the financial and corporate contexts, I demonstrate why that groundwork is incompatible with the AR4D context, and replace it with suitable foundations. More specifically, in place of strained arguments that would construe project NPV as a traded good in an efficient market, I show how a tractable multistage ROV model for AR4D research products follows from the definition of NPV itself, and from the assumption that the time evolution of project NPV follows a geometric Brownian motion (gBm). Because the gBm model is widely eschewed in the ROV literature, the main derivation of the proposed multistage model is preceded by a substantial defense of this methodological choice. To illustrate the proposed method’s utility, the main derivation is followed by a hypothetical use case to evaluate the ROV of stage 1 research of a real 4-stage AR4D project (Late Blight resistant potato).

It is important to distinguish the work presented here from a separate “real options approach” that examines the optimal delay, from a social planner’s perspective, of releasing a completed transgenic agricultural R&D product under environmental uncertainty [21–24]. That line of work builds on the American-style option-to-delay framework developed by Dixit and Pindyck [3] in conjunction with the concept of “quasi-option value” developed in environmental economics [25]. By contrast, the present work concerns the valuation of sequential, stage-gated investments in research that has yet to be undertaken, modeled as European-style options with fixed-horizon investment cycles, as formalized in Eq (1).

## 2. The incompatible financial underpinnings of existing ROV approaches

### 2.1 AR4D products are not traded goods in efficient markets

The ROV literature cited above has developed in the context of corporate projects aiming to develop new goods or services to be marketed for a profit. In such a market or near-market setting, a financial argument known as “replicating portfolios” may be invoked [26–28]. Methodologically, the replicating portfolios argument means that corporate project NPV is well approximated (“replicated”) by a linear combination of relevant prices. This greatly facilitates the estimation of project NPV and risk—both of which are required for ROV—as they may be calculated at any time from publicly available price information.

AR4D projects, on the other hand, are born of market failures, where no such argument may be invoked; and so AR4D project NPV and risk must be calculated the “hard way”, by ex-ante impact assessment. Some might contend that the recent emergence of carbon and green bond markets [29,30] paves the way for a replicating portfolios analogue in the AR4D context. However, in addition to green outcomes, AR4D projects typically focus on health, nutrition, and socioeconomic outcomes for marginalized populations, the respective values of which, almost by definition, are not reflected in any market. And, even if they were—even if there were an “SDG bond” mapped to every AR4D impact area—this might be useful in the evaluation of project NPV, but of very little use in the assessment of project risk, since the main source of AR4D risk is not “price risk” (tragically, there is little risk of the bottom falling out of the SDG “market” in our lifetimes), but rather technical risk, i.e., the risk that the proposed technology will not function or scale as expected, due either to technical difficulties encountered in the research itself, or to unforeseen adversity in the enabling environment (e.g., government policy shifts, deregulation issues, etc.).

Even in the corporate ROV context, the replicating portfolios argument is met with considerable skepticism [31]; and proponents of the argument acknowledge that its validity is limited to a narrow range of project types, which generally does not include R&D projects [32]. There is strong methodological incentive to invoke the argument because the fundamental equation governing all types of option (and all financial derivative) valuation—i.e., the Black-Scholes partial differential equation (PDE)—is formally derived from an assumption in financial markets known as the “no-arbitrage rule”, which requires that the underlying project NPV be interpretable as a traded good in an efficient market [19].

To preserve the Black-Scholes PDE in cases where the replicating portfolios argument may not be invoked, some corporate ROV practitioners invoke the “complete markets” assumption as a second-best alternative [32–34]. Under the complete markets assumption, project NPV cannot be modeled as a linear function of prices, but the Black-Scholes PDE is nonetheless preserved because “all risks can be hedged by trading securities” [34]. As just mentioned, the main source of AR4D project risk is technical risk associated with non-market factors. Clearly, then, AR4D risk cannot be hedged by trading securities; and so there are no grounds in the AR4D context on which to invoke complete markets.

More problematically still, the formal derivation of the Black-Scholes PDE from either the replicating portfolios or complete markets assumptions leads to a celebrated corollary known as “risk-neutral valuation”, which implies that “investors do not increase the expected return they require from an investment in order to compensate for increased risk” [19]. Clearly, AR4D donors are not risk neutral. They are willing to bet on a high risk project only if there is a sufficiently high expected return on the investment.

In the derivation of the AR4D ROV formula presented farther below, these problematic foundations are replaced with the comparatively simple assumption that future values of project NPV are lognormally distributed. The derivation also requires that the expected growth rate of project NPV be equal to the discount rate, but this follows trivially from the definition of project NPV itself, and thus does not have to be assumed.

For present purposes, the assumption of lognormally distributed project NPV is tantamount to assuming that project NPV evolves as a gBm, a notion that is eschewed in the financial and corporate contexts in favor of more complex stochastic models [35,36]. Before proceeding to the derivation of the multistage AR4D ROV formula, then, it is first necessary to introduce, contextualize, and defend the choice of gBm as a suitable model of the time evolution of project NPV.

### 2.2 In defense of gBm as a reasonable model of the time evolution of AR4D project NPV

**Fig 1:**
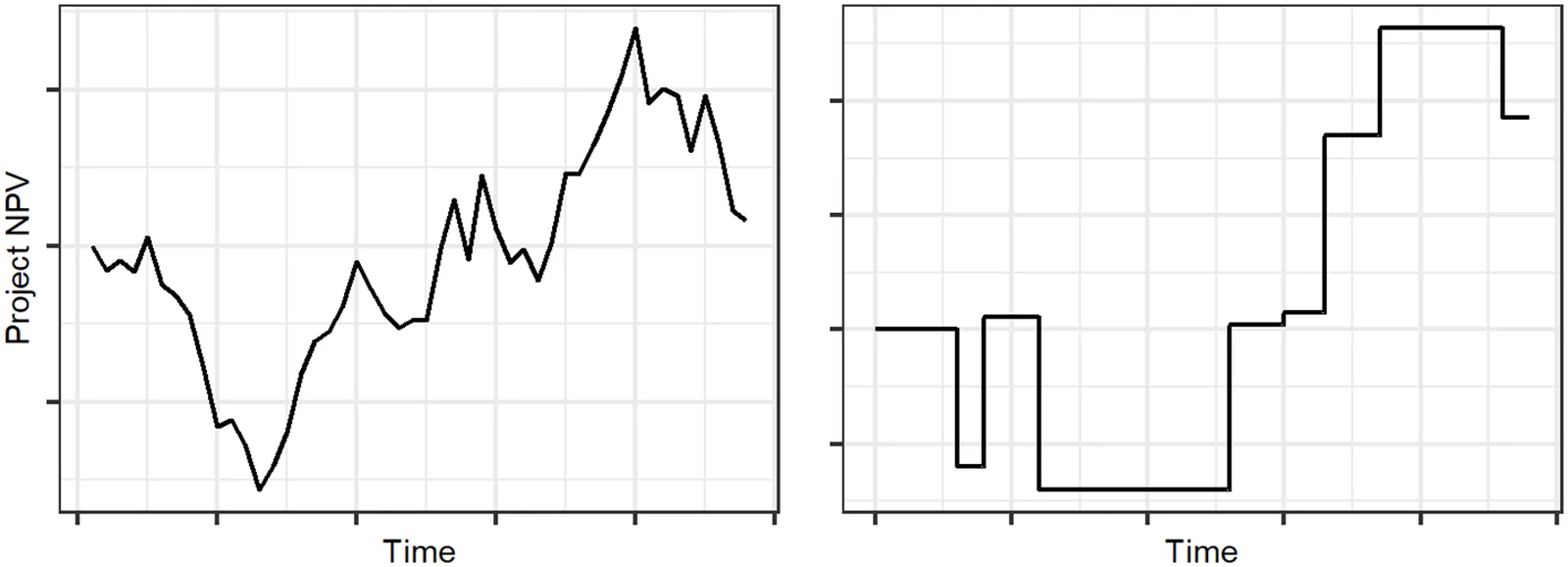
Examples of project NPV evolving over quarterly time steps. (Left) As a geometric Brownian motion and (right) a compound Poisson process.

In settings such as AR4D where project NPV time series data are not readily available, a suitable choice of stochastic model of the time evolution of project NPV may nonetheless be guided deductively by consideration of the size distribution of future values of NPV, or the size distribution of changes in NPV per relevant small time increment (e.g., an annual quarter in the context of 10–20 year AR4D projects). Changes in project NPV over such intervals are driven by changes in underlying component quantities—the probability that a research target is achieved, the likely rate of technology adoption, the estimated magnitude of welfare impacts on target populations, and so forth. These changes operate multiplicatively on project value. A change in adoption potential, for instance, scales the entire expected benefit stream upward or downward by some factor rather than adding or subtracting a fixed amount. This multiplicative structure implies that proportional changes in NPV—equivalently, changes in log NPV—are the natural unit of analysis, rather than absolute changes in NPV levels. When such proportional changes are individually small, collectively numerous, and approximately independent, log NPV changes over small time increments may plausibly be approximated as normally distributed. Normally distributed log NPV changes, in turn, are the distributional signature of a gBm process. The left panel of Fig 1 depicts a simulated gBm trajectory generated through the accumulation of such proportional changes.

In the financial context, the gBm model is eschewed as unrealistic because it does not reflect the abrupt jumps empirically observed in many price series, which are inconsistent with a lognormal size distribution. Instead, “jump” or “jump-diffusion” models are recommended, which more accurately capture these abrupt swings in value [35,36]. An example of a jump process, known as the compound Poisson process (cPp), is displayed in the right panel of Fig 1 (algorithm 6.2 in Cont and Tankov [36]).

The gBm model is methodologically attractive because it allows for a closed form solution to the Black-Scholes PDE. The greater financial realism of jump and jump diffusion models, on the other hand, comes at the high methodological cost of requiring numerical solutions to the Black-Scholes PDE. Corporate ROV methods have adopted the financial context’s consensus against gBm in favor of jump and jump diffusion models, thus inheriting the same high degree of methodological complexity. Trigeorgis once characterized this complexity as the unavoidable “bitter pill” that all serious ROV practitioners must come to grips with [37].

In hindsight, the appetite for bitterness outside of academic audiences—that is to say, adoption of ROV thinking by real world decision makers—has remained low [6,38–41]. Both critics and proponents alike attribute this tepid reception to the formal complexity of ROV, much of which can be traced back to the use of sophisticated stochastic models and numerical methods [39,41]. From the perspective of research donors and managers, such techniques are effectively black boxes; and even ROV experts find themselves challenged at times. A much cited numerical exercise by Majd and Pindyck [33], for example, was later shown to contain an elementary error [42]. It is not always clear, moreover, what is gained in exchange for the bitterness; or whether one set of questionable assumptions is merely replaced with another. The cPp model (e.g., as specified by Pennings and Lint [34]) rests on the assumption that changes in project NPV occur at random intervals [36]. Yet substantive reassessments of project NPV typically occur at regular intervals—e.g., through scheduled reviews, milestone evaluations, reporting cycles, or electoral calendars.

This observation about reporting intervals, in turn, points to a deeper consideration that is particularly consequential in the AR4D context: discrete jumps in project NPV are often artifacts of reporting protocols, reflecting not changes in value as they occur, but rather moments when the cumulative effect of a series of such changes is finally made known through a discrete report. This filtration logic applies to both internal management updates and external macro-events. An unexpected government policy shift or field trial result registers as a discontinuous jump only because the continuous, inscrutable processes leading up to it—unobserved ecological dynamics, closed-door political deliberations, gradually shifting coalitions and preferences—remain hidden from view until a formal decree or scheduled measurement and report.

It bears emphasis that this observation extends beyond ecology, where it is relatively obvious, to also encompass social, political, and institutional processes, which are typically experienced as a series of discrete threshold events—collective decisions, policy enactments, norm changes, institutional reforms, and the like. Underlying the apparently discontinuous character of such processes there exists a latent process that may plausibly be treated as continuous. That is to say, the vote is binary, but the deliberation, coalition-building, and preference evolution leading up to it are not; the policy announcement is instantaneous, but the political economy generating it evolves gradually; the institution appears to change abruptly, but the accumulation of contradictions between its formal rules and informal practices, and the gradual realignment of stakeholder interests that precipitate reform are continuous processes operating beneath the surface of apparent stability [43].

It is therefore reasonable to distinguish between a process as reported, which may exhibit discontinuities as an artifact of low-frequency sampling and coarse measurement, and the latent process that is reported, which evolves continuously and independently of the reporting protocol. This continuity becomes still more plausible in the case of aggregate trajectories such as project NPV, which integrate numerous heterogeneous ecological, technical, political, economic, organizational, and social processes.

In the financial and corporate research contexts, this distinction may be largely irrelevant since the publicly reported value of company activities, as reflected in stock prices, is itself the economically relevant object of interest, regardless of what the firm’s “true” value may be when insider or otherwise unobservable information is taken into account. In the AR4D setting, however, there is no analogous need to accommodate the filtration structure imposed by measurement and reporting conventions. For purposes of ex-ante impact assessment, the analyst is concerned with the latent stochastic evolution of the project’s expected social value itself, regardless of when or how or even if the value is observed and reported. Once this distinction is made, the empirical justification for modeling AR4D project value through jump processes becomes substantially weaker—and the case for a continuous diffusion model correspondingly stronger. Insofar as project NPV reflects the cumulative effect of many small, approximately independent proportional influences of finite variance, functional central limit theorems imply that, as the reporting interval shrinks toward zero, the log-value process may be approximated by Brownian motion, and hence project value by gBm [44,45].

A separate objection is that AR4D project value may be path dependent—in the sense of David [46] and Arthur [47]—whereby historical sequences of events influence future technological and institutional trajectories, a concern that Ruttan [48] discusses in the context of agricultural innovation. While this literature primarily characterizes industry- and system-level dynamics, it raises the concern that project/asset level Markovian valuation models such as gBm may be misspecified if current NPV does not constitute a sufficient summary of past developments, and therefore may fail to capture the effects of mechanisms such as technological lock-in on future value. This concern applies not only to gBm but to all Markovian models of value dynamics, including the jump and jump-diffusion processes just discussed. Nor is it specific to non-market research contexts. The path dependence literature itself originated in analyses of technological adoption in the private sector [46,47], where firm value is likewise shaped by early adoption dynamics, network externalities, and related lock-in mechanisms.

Financial economics has long acknowledged the role of such historical contingencies in shaping fundamentals, and yet asset prices are routinely modeled using Markovian approximations. This is because the canonical efficient markets literature [49–51] shows that, under rational expectations and sufficiently strong incentives for valuation errors to be corrected, current prices incorporate available information about underlying fundamentals such that past price movements do not provide systematic predictive power. While this martingale characterization does not itself assert that price is a sufficient summary state variable, subsequent practice operationalizes this insight by specifying Markov models of asset value evolution [19,35,52], which effectively treat current price as a sufficient summary—at least for practical decision-making purposes—of the information contained in past developments.

This practical implication—that current value adequately summarizes decision-relevant historical information despite path dependent fundamentals—rests on two complementary premises. The first, formalized in Samuelson’s [49] axiom of mathematically expected price formation, holds that if a price were known with certainty to rise tomorrow, it would have already risen today. That is to say, rational agents immediately incorporate any predictable future change into current value, leaving only genuinely new information to drive future revisions. The second, central to Fama’s [53] empirical argument, is the existence of “sophisticated traders” who continuously scrutinize assets for discrepancies between market price and their own expert assessments of fundamental value, and act to eliminate such discrepancies. Together, these premises enforce the martingale property of current value and underpin the practical adequacy of Markovian approximations. The widespread use of such models therefore reflects not a denial of historical contingency, but a consequence of these rationality conditions, under which the informational consequences of historical contingency are incorporated into current value.

Both of these canonical premises are present in the non-market AR4D setting, if in attenuated form. A rational ex-ante impact assessor of an AR4D project, like Samuelson’s price-forming agent, will immediately revise their current NPV estimate upward if they know with certainty that project value will rise. To do otherwise would constitute a straightforward violation of rational expectation formation. And ex-ante impact assessors are, by professional function, precisely analogous to Fama’s sophisticated traders: they conduct discrete, independent expert assessments of a project value trajectory shaped by the same class of fundamental processes (economic, political, institutional, environmental, etc.), and revise current estimates whenever their analysis reveals a discrepancy. The essential difference between market and non-market settings therefore lies in the discovery mechanism, not in the nature of the latent value process being discovered. More specifically, the difference is one of sampling rate and resolution. A liquid asset market aggregates large numbers of independent expert assessments per second, washing out individual analyst bias and approximating the martingale ideal at high fidelity. An AR4D impact assessment approximates an analogous latent value, shaped by the same class of historically conditioned fundamentals, at far coarser resolution—periodically rather than continuously, through few analysts rather than many, with weaker incentive-based bias correction.

Even if one grants that substantial discontinuities or path-dependent effects may occasionally arise over the course of an AR4D project’s 10–20 year trajectory, the practical significance of this concern is substantially mitigated by the multistage structure of the proposed decision model. The relevant valuation problem is not the direct appraisal of a single uninterrupted multi-decade payoff stream, but rather the sequential valuation of continuation options over comparatively short decision intervals, typically on the order of 1–5 years in the AR4D context. At each stage, updated information is incorporated into revised continuation values, which then serve as the state variables governing subsequent decisions. The operational question is therefore not whether the entire long-run trajectory is perfectly continuous and Markovian, but whether project value may reasonably be approximated as locally continuous and Markovian between adjacent decision nodes after information updating. This is a substantially weaker requirement.

Finally, it should be borne in mind that the choice of a stochastic model for project NPV does not constitute a claim that the selected model provides a literal description of reality. In ex-ante AR4D appraisal, the latent evolution of project value is not directly observable, and competing specifications can rarely be ranked through decisive empirical tests. The choice is therefore not between a correct model and incorrect alternatives, but among imperfect approximations that differ in their conceptual plausibility, analytical tractability, transparency to non-specialist users, and usefulness for guiding sequential investment decisions. Evaluated on these criteria, gBm occupies a defensible and arguably privileged position. It is theoretically motivated by functional central limit theorem arguments (as discussed above), consistent with the martingale characterization of rationally updated valuations, free of the information filtration assumptions that burden jump-based alternatives in non-market settings, and sufficiently tractable to remain interpretable to the research donors and managers whose decisions it is ultimately designed to inform.

## 3. Formal derivation of multistage ROV in the AR4D context

### 3.1 Derivation of single-stage ROV

Assuming *x*(*t*) follows a gBm, the evolution Δx over a small time increment Δt is described by


$$\begin{array}{c} \Delta x=x(t)m\Delta t+x(t)s\epsilon \sqrt{\Delta t}\;\;;\;\;\;\Delta x:=x(t+\Delta t)-x(t) \end{array}$$ (8)

Where ϵ is a normally distributed random variable with mean 0 and variance 1, such that *m* and *s* quantify the trend and volatility of the time evolution, respectively.


$$\begin{array}{cc} m\Delta t & =E\left[\frac{\Delta x}{x}\right]\\ s^{2}\Delta t & =Var\left[\frac{\Delta x}{x}\right] \end{array}$$ (9)

In other words, mΔt and $s^{2}\Delta t$ are the mean and variance of the instantaneous arithmetic return or growth rate. The expected value of project NPV at some future time *T* as evaluated at time *t* = 0 is given by


$$\begin{array}{c} E[x(T)]{|}_{t=0}=e^{mT}x(0) \end{array}$$ (10)

And the mean and variance of the log return over any finite interval [0, *T*] are given by


$$\begin{array}{c} E\left[\ln\left(\frac{x(T)}{x(0)}\right)\right]{|}_{t=0}=\left(m-\frac{s^{2}}{2}\right)T \end{array}$$ (11)

$$\begin{array}{c} Var\left[\ln\left(\frac{x(T)}{x(0)}\right)\right]{|}_{t=0}=s^{2}T \end{array}$$ (12)

(See Hull [19] for details.)

Since the calculation of project NPV already accounts for all expected changes to NPV that might occur over the time horizon, then, by definition, the expected growth rate of project NPV is just the discount rate (*m* = *r*).

The single-stage ROV formula follows by evaluating Eq (1) through straightforward integration.

In general, for a lognormally distributed random variable *q* and a constant *C*,


$$\begin{array}{c} E[\max(q-C,0)]=E[q]\Phi \left(\frac{\ln\left(\frac{E[q]}{C}\right)+\frac{\omega^{2}}{2}}{\omega }\right)-C\Phi \left(-\frac{\ln\left(\frac{E[q]}{C}\right)-\frac{\omega^{2}}{2}}{\omega }\right) \end{array}$$ (13)

Where Φ() is the standard cumulative normal distribution function. (See S1 Appendix for proof.)

In the case where *q* = *x*(*T*_0_), *C* = *K*_0_, and multiplying through by the discount factor $e^{-rT_{0}}$, this becomes


$$\begin{array}{c} e^{-rT_{0}}E[\max(x(T_{0})-K_{0},0)]{|}_{t=0}=x(0)\Phi \left(\delta_{0}+s\sqrt{T_{0}}\right)-e^{-rT_{0}}K_{0}\Phi (\delta_{0}) \end{array}$$ (14)

Where


$$\begin{array}{c} \delta_{0}=\frac{\ln\left(\frac{x(0)}{K_{0}}\right)+\left(r-\frac{s^{2}}{2}\right)T_{0}}{s\sqrt{T_{0}}} \end{array}$$ (15)

Note that the value of $\Phi (\delta_{0})$ is instructive. It is the probability that *x*(*T*_0_) will exceed the exercise cost *K*_0_—or, in real options parlance, the probability that the option finishes “in the money”. To see this, note that $\delta_{0}$ may be rewritten as the negative standard score of $\ln(K_{0}/x(0))$.


$$\begin{array}{cc} \delta_{0} & =-\frac{\ln\left(\frac{K_{0}}{x(0)}\right)-\left(r-\frac{s^{2}}{2}\right)T_{0}}{s\sqrt{T_{0}}}\\ & =-\frac{\ln\left(\frac{K_{0}}{x(0)}\right)-E\left[\ln\left(\frac{x(T_{0})}{x(0)}\right)\right]{|}_{t=0}}{s\sqrt{T_{0}}} \end{array}$$ (16)

Hence,


$$\begin{array}{cc} \Phi (\delta_{0}) & =1-\Phi \left(\frac{\ln\left(\frac{K_{0}}{x(0)}\right)-E\left[\ln\left(\frac{x(T_{0})}{x(0)}\right)\right]{|}_{t=0}}{s\sqrt{T_{0}}}\right)\\ & =1-P\left(\ln(x(T_{0}))\leq \ln(K_{0})\right)\\ & =P\left(\ln(x(T_{0}))\geq \ln(K_{0})\right) \end{array}$$ (17)

### 3.2 Derivation via the Itô calculus and relation to the Black-Scholes derivation

Geometric Brownian motion is a particular case of a broader class of stochastic processes known as Itô processes, defined as follows.


$$\begin{array}{c} \Delta x=a(x,t)\Delta t+b(x,t)\epsilon \sqrt{\Delta t} \end{array}$$ (18)

Where *a* and *b* are functions of *x* and *t*.

Itô’s lemma states that the evolution of a function *f*(*x*, *t*) is also an Itô process, described as follows [19].


$$\begin{array}{c} \Delta f=\left(\frac{\partial f}{\partial x}a+\frac{\partial f}{\partial t}+\frac{1}{2}\frac{\partial^{2}f}{\partial x^{2}}b^{2}\right)\Delta t+\frac{\partial f}{\partial x}b\epsilon \sqrt{\Delta t} \end{array}$$ (19)

In the present case, where *x*(*t*) is a gBm with *a* = *x r*, *b* = *x s*, Itô’s lemma reduces to


$$\begin{array}{c} \Delta f=\left(\frac{\partial f}{\partial x}xr+\frac{\partial f}{\partial t}+\frac{s^{2}x^{2}}{2}\frac{\partial^{2}f}{\partial x^{2}}\right)\Delta t+\frac{\partial f}{\partial x}xs\epsilon \sqrt{\Delta t} \end{array}$$ (20)

It follows that the expected value and variance of the instantaneous growth rate of *f* are, respectively,


$$\begin{array}{c} E\left[\frac{\Delta f}{f}\right]=\frac{1}{f}\left(\frac{\partial f}{\partial x}xr+\frac{\partial f}{\partial t}+\frac{s^{2}x^{2}}{2}\frac{\partial^{2}f}{\partial x^{2}}\right)\Delta t \end{array}$$ (21)

and


$$\begin{array}{c} Var\left[\frac{\Delta f}{f}\right]=\eta_{1,0}^{2}s^{2}\Delta t \end{array}$$ (22)

Where the shorthand $\eta_{1,0}=\frac{x}{f}\frac{\partial f}{\partial x}$ has been introduced. (See Hull [19] for details.)

The function *f*(*x*, *t*) in mind here is, of course, ROV (Eq (14)). Because ROV is defined as an expected future value discounted back to the present, then its expected instantaneous growth rate is, by definition, E[Δf/f]=rΔt. Eq (21) may thus be set equal to rΔt, resulting in the following PDE.


$$\begin{array}{c} fr=\frac{\partial f}{\partial x}xr+\frac{\partial f}{\partial t}+\frac{s^{2}x^{2}}{2}\frac{\partial^{2}f}{\partial x^{2}} \end{array}$$ (23)

This may then be solved for *f* under the boundary condition $f(T_{0})=\max(x(T_{0})-K_{0},0)$, resulting in the same expression obtained through straightforward integration in Eq (14). (See S1 Appendix for details.)

To reach the same result in the financial context, Black and Scholes [52] famously noted that Eqs (8) and 20 could be combined so as to eliminate the random term as follows.


$$\begin{array}{c} \Delta f-\frac{\partial f}{\partial x}\Delta x=\left(\frac{\partial f}{\partial t}+\frac{s^{2}x^{2}}{2}\frac{\partial^{2}f}{\partial x^{2}}\right)\Delta t \end{array}$$ (24)

In the financial context, the left-hand side of this equation may be thought of as the instantaneous evolution over the increment Δt of a portfolio long one share of the financial derivative *f* and short a quantity ∂f/∂x of the underlying security *x*(*t*). This is where Black and Scholes applied their no-arbitrage argument: Since the random—i.e., risky—term has been eliminated from Eq (24), then *t*he profit or loss of this portfolio over the increment Δt must be riskless. That is, it must change at the risk free rate *r*.


$$\begin{array}{c} \Delta f-\frac{\partial f}{\partial x}\Delta x=\left(f-\frac{\partial f}{\partial x}x\right)r\Delta t \end{array}$$ (25)

Equating the right-hand side of this equation with the right-hand side of the previous equation, the Δt’s cancel, resulting in Eq (23).

Note that the Black-Scholes derivation hinges critically upon the assumption that *x*(*t*) follows a gBm. As discussed in the previous section, this assumption is largely rejected in the literature subsequent to Black and Scholes’ seminal paper, thus rendering the closed form solution in Eq (14) of mere academic interest in the financial and corporate ROV contexts.

To tidy up, note that, since the expected growth rate of ROV is defined as rΔt, then the time evolution of *f* in Eq (20) may be rewritten more simply as follows.


$$\begin{array}{c} \Delta f=fr\Delta t+f\eta_{1,0}s\epsilon \sqrt{\Delta t} \end{array}$$ (26)

A further consequence of Itô’s lemma is that log changes in *f* are normally distributed with mean and variance


$$\begin{array}{c} E\left[\ln\left(\frac{f(T_{1})}{f(0)}\right)\right]{|}_{t=0}=\left(r-\eta_{1,0}^{2}\frac{s^{2}}{2}\right)T_{1} \end{array}$$ (27)

$$\begin{array}{c} Var\left[\ln\left(\frac{f(T_{1})}{f(0)}\right)\right]{|}_{t=0}=\eta_{1,0}^{2}s^{2}T_{1} \end{array}$$ (28)

While the Itô calculus is not necessary for deriving the single-stage ROV formula (this was demonstrated in the previous subsection), it becomes useful in the derivation of a formula for multistage ROV because it provides a clear demonstration that ROV itself is lognormally distributed with computable parameters given in Eqs (27) and (28); and that this is recursively true for the ROV of *f*, and for the ROV of the ROV of *f*, and so on, ad infinitum, as detailed in the next section.

### 3.3 Extension to multistage ROV

For the derivation of a multistage ROV formula, it is henceforth necessary to index *f* as *f*_1_, *f*_2_,..., on up to $f_{n}$. (The $f_{i}$ correspond to the $ROV_{i}$ appearing in the Introduction). Following this schema, *f*_1_ corresponds to Eq (14) and to *f* in the equations above, and *f*_0_ corresponds to project NPV *x*.

Because *f*_1_ is itself lognormally distributed, the same formula used to evaluate *f*_1_ can be used to evaluate $f_{2}(T_{2})$. That is to say, Eq (13) may be evaluated at $q=f_{1}(T_{1})$ and *C* = *K*_1_ to calculate *f*_2_, the value of the option to invest in the last stage of research.


$$\begin{array}{cc} f_{2}(f_{1},t;T_{1},K_{1},s,r){|}_{t=0} & =e^{-rT_{1}}E[\max(f(T_{1})-K_{1},0)]{|}_{t=0}\\ & =f_{1}(0)\Phi (\delta_{1}+\eta_{1,0}s\sqrt{T_{1}})-e^{-rT_{1}}K_{1}\Phi (\delta_{1}) \end{array}$$ (29)

Where *T*_1_ is the time until completion of stage 1, *K*_1_ is the research investment required upon successful completion of stage 1—where stage 1 is considered successfully completed if $f_{1}(T_{1})>K_{1}$—and where


$$\begin{array}{c} \delta_{1}=\frac{\ln\left(\frac{f_{1}(0)}{K_{1}}\right)-\left(r-\eta_{1,0}^{2}\frac{s^{2}}{2}\right)T_{1}}{\eta_{1,0}s\sqrt{T_{1}}} \end{array}$$ (30)

Moreover, because *f*_1_ is an Itô process and *f*_2_ is a function of *f*_1_, then by Itô’s lemma, *f*_2_ is also an Itô process,


$$\begin{array}{c} \Delta f_{2}=\left(\frac{\partial f_{2}}{\partial f_{1}}rf_{1}+\frac{\partial f_{2}}{\partial t}+\eta_{1,0}^{2}\frac{s^{2}f_{1}^{2}}{2}\frac{\partial^{2}f_{2}}{\partial f_{1}^{2}}\right)\Delta t+f_{2}\eta_{2,0}s\epsilon \sqrt{\Delta t} \end{array}$$ (31)

With expected value and variance of the instantaneous growth rate of *f*_2_


$$\begin{array}{c} E\left[\frac{\Delta f_{2}}{f_{2}}\right]=\frac{1}{f_{2}}\left(\frac{\partial f_{2}}{\partial f_{1}}rf_{1}+\frac{\partial f_{2}}{\partial t}+\eta_{1,0}^{2}\frac{s^{2}f_{1}^{2}}{2}\frac{\partial^{2}f_{2}}{\partial f_{1}^{2}}\right)\Delta t \end{array}$$ (32)

$$\begin{array}{c} Var\left[\frac{\Delta f_{2}}{f_{2}}\right]=\eta_{2,0}^{2}s^{2}\Delta t\;\;;\;\;\;\eta_{2,0}=\frac{f_{1}}{f_{2}}\frac{\partial f_{2}}{\partial f_{1}}\eta_{1,0} \end{array}$$ (33)

And such that the log return $\ln(f_{2}(T)/f_{2}(t))$ for some future time $T\in (0,T_{1})$ is normally distributed with expected value and variance


$$\begin{array}{cc} E\left[\ln\left(\frac{f_{2}(T)}{f_{2}(t)}\right)\right]{|}_{t=0} & =\left(r-\eta_{2,0}^{2}\frac{s^{2}}{2}\right)T\\ Var\left[\ln\left(\frac{f_{2}(T)}{f_{2}(t)}\right)\right]{|}_{t=0} & =\eta_{2,0}^{2}s^{2}T \end{array}$$ (34)

Whereby it follows that the same formula used to evaluate the ROV of $f_{1}(T_{1})$ and $f_{2}(T_{2})$ can be used to evaluate the ROV of $f_{3}(T_{3})$. That is to say, Eq (13) may be evaluated at $q=f_{2}(T_{2})$ and *C* = *K*_2_ to calculate *f*_3_, the value of the option to invest in the second to last stage of research.


$$\begin{array}{cc} f_{3}(f_{2},t;T_{2},K_{2},s,r){|}_{t=0} & =e^{-rT_{2}}E[\max(f(T_{2})-K_{2},0)]{|}_{t=0}\\ & =f_{2}(0)\Phi \left(\delta_{2}+\eta_{2,0}s\sqrt{T_{2}}\right)-e^{-rT_{2}}K_{2}\Phi (\delta_{2}) \end{array}$$ (35)

Where *T*_2_ is the time until completion of stage 1, *K*_2_ is the research investment required upon successful completion of stage 1—where stage 1 is considered successfully completed if $f_{2}(T_{2})>K_{2}$—and where


$$\begin{array}{c} \delta_{2}=\frac{\ln\left(\frac{f_{2}(0)}{K_{2}}\right)-\left(r-\eta_{2,0}^{2}\frac{s^{2}}{2}\right)T_{2}}{\eta_{2,0}s\sqrt{T_{2}}} \end{array}$$ (36)

And so on and so forth, it follows generally that the ROV of stage 1 of any *n*-stage project, $f_{n}$, is an Itô process,


$$\begin{array}{c} \Delta f_{n}=\left(\frac{\partial f_{n}}{\partial f_{n-1}}rf_{n-1}+\frac{\partial f_{n}}{\partial t}+\eta_{n-1,0}^{2}\frac{s^{2}f_{n-1}^{2}}{2}\frac{\partial^{2}f_{n}}{\partial f_{n-1}^{2}}\right)\Delta t+f_{n}\eta_{n,0}s\epsilon \sqrt{\Delta t} \end{array}$$ (37)

And is thus lognormally distributed with parameters


$$\begin{array}{c} \begin{array}{c} E\left[\ln\left(\frac{f_{n}(T)}{f_{n}(t)}\right)\right]{|}_{t=0}=\left(r-\eta_{n,0}^{2}\frac{s^{2}}{2}\right)T\\ Var\left[\ln\left(\frac{f_{n}(T)}{f_{n}(t)}\right)\right]{|}_{t=0}=\eta_{n,0}^{2}s^{2}T \end{array}\;\;;\;\;\;0<T<T_{n-1} \end{array}$$ (38)

Such that a general formula for the ROV of stage 1 of any *n*-stage project may be resolved by evaluating Eq (13) at $q=f_{n-1}(T_{n-1})$, $C=K_{n-1}$, giving


$$\begin{array}{cc} f_{n}(f_{n-1},t;T_{n-1},K_{n-1},s,r){|}_{t=0} & =e^{-rT_{n-1}}E[\max(f_{n-1}(T_{n-1})-K_{n-1},0)]{|}_{t=0}\;\;;\;\;\;n\in {\mathbb{N}}_{0}\\ & =f_{n-1}(0)\Phi \left(\delta_{n-1}+\eta_{n-1,0}s\sqrt{T_{n-1}}\right)-e^{-rT_{n-1}}K_{n-1}\Phi (\delta_{n-1}) \end{array}$$ (39)

Where $T_{n-1}$ is the time until completion of stage 1 of the *n*-stage research project, $K_{n-1}$ is the research investment required upon successful completion of stage 1 at time $T_{n-1}$, where stage 1 is considered successfully completed if $f_{n-1}(T_{n-1})>K_{n-1}$, and where


$$\begin{array}{c} \delta_{n-1}=\frac{\ln\left(\frac{f_{n-1}(0)}{K_{n-1}}\right)-\left(r-\eta_{n-1,0}^{2}\frac{s^{2}}{2}\right)T_{n-1}}{\eta_{n-1,0}s\sqrt{T_{n-1}}} \end{array}$$ (40)

The ROV of stage 1 of any *n*-stage project is thus calculated recursively, starting with the calculation of the ROV of the last stage $f_{1}(x,t;T_{0},K_{0},s,r){|}_{t=0}$, which then feeds into the calculation of the ROV of the second to last stage, $f_{2}(f_{1},t;T_{1},K_{1},s,r){|}_{t=0}$, and so forth until the calculation of first stage ROV $f_{n}(f_{n-1},t;T_{n-1},K_{n-1},s,r){|}_{t=0}$. The ROV $f_{i}$ calculated at each stage is the value of the option to invest in the subsequent stage.


$$\begin{array}{cc} f_{i}(f_{i-1},t;T_{i-1},K_{i-1},s,r){|}_{t=0} & =e^{-rT_{i-1}}E[\max(f_{i-1}(T_{i-1})-K_{i-1},0)]{|}_{t=0}\\ & =f_{i-1}(0)\Phi \left(\delta_{i-1}+\eta_{i-1,0}s\sqrt{T_{i-1}}\right)-e^{-rT_{i-1}}K_{i-1}\Phi (\delta_{i-1}) \end{array}$$ (41)

$$\begin{array}{c} \delta_{i-1}=\frac{\ln\left(\frac{f_{i-1}(0)}{K_{i-1}}\right)-\left(r-\eta_{i-1,0}^{2}\frac{s^{2}}{2}\right)T_{i-1}}{\eta_{i-1,0}s\sqrt{T_{i-1}}} \end{array}$$ (42)

In this process, the respective probabilities of finishing in the money (project stage success), $\Phi (\delta_{i})$, and elasticities of $f_{i}$ with respect to project NPV, $\eta_{i,0}$, are also calculated for each stage. The elasticity $\eta_{i,0}$ is an instructive measure of ROV sensitivity to changes in project NPV. It is calculated for each project stage i∈[0,n] as follows.


$$\begin{array}{c} \eta_{i,0}=\frac{f_{i-1}}{f_{i}}\frac{\partial f_{i}}{\partial f_{i-1}}\eta_{i-1,0}\;\;;\;\;\;0\leq i\leq n\;\;\;\eta_{0,0}=1 \end{array}$$ (43)

Where the derivative $\partial f_{i}/\partial f_{i-1}$ follows from Eq (41).


$$\begin{array}{c} \frac{\partial f_{i}}{\partial f_{i-1}}=\Phi \left(\delta_{i-1}+\eta_{i-1,0}s\sqrt{T_{i-1}}\right)+s\sqrt{T_{i-1}}\frac{\partial \eta_{i-1,0}}{\partial f_{i-1}}f_{i-1}\phi \left(\delta_{i-1}+\eta_{i-1,0}s\sqrt{T_{i-1}}\right) \end{array}$$ (44)

Where ϕ() is the standard normal probability density function, and


$$\begin{array}{c} \frac{\partial \eta_{i-1,0}}{\partial f_{i-1}}=\left\{ \begin{array}{cc} \frac{\partial }{\partial f_{i-1}}1=0 & ,\;\;\;i=1\\ \frac{\partial }{\partial f_{i-1}}\left(\frac{f_{i-2}}{f_{i-1}}\frac{\partial f_{i-1}}{\partial f_{i-2}}\right)=-\frac{\eta_{i-1}}{f_{i-1}} & ,\;\;\;i>1 \end{array}\right. \end{array}$$ (45)

Finally, note that the multistage ROV formula may also be obtained in a “Black-Scholes manner” by recognizing that $f_{n}$ is defined as an expected future value discounted back to the present, such that the expected growth rate of $f_{n}$ is, by definition, just $E[\Delta f_{n}/f_{n}]=r\Delta t$. Since $f_{n}$ is an Itô process, then the expected growth rate is also given by


$$\begin{array}{c} E\left[\frac{\Delta f_{n}}{f_{n}}\right]=\frac{1}{f_{n}}\left(\frac{\partial f_{n}}{\partial f_{n-1}}rf_{n-1}+\frac{\partial f_{n}}{\partial t}+\eta_{n-1,0}^{2}\frac{s^{2}f_{n-1}^{2}}{2}\frac{\partial^{2}f_{n}}{\partial f_{n-1}^{2}}\right)\Delta t \end{array}$$ (46)

Setting this equal to rΔt results in a generalized Black-Scholes PDE for $f_{n}$,


$$\begin{array}{c} rf_{n}=f_{n-1}r\frac{\partial f_{n}}{\partial f_{n-1}}+\frac{\partial f_{n}}{\partial t}+\eta_{n-1,0}^{2}\frac{s^{2}f_{n-1}^{2}}{2}\frac{\partial^{2}f_{n}}{\partial f_{n-1}^{2}} \end{array}$$ (47)

Which may then be solved for $f_{n}$ under the boundary condition $f_{n}(T_{n})=\max(f_{n-1}(T_{n-1})-K_{n-1},0)$ as another way of obtaining Eq (39).

## 4. An illustrative example

As an illustrative example, the multistage ROV formula developed above is applied to calculate the ROV a real multistage AR4D project currently in implementation. The aim of the project in question is to develop a transgenic potato variety with resistance to Late Blight disease (henceforth LBr potato), for release as a public good in two developing countries. Late Blight is the disease behind the infamous Irish Potato Famine of 1845–1849; and continues to pose a major threat to food security, especially in the developing world [54,55]. Successful research and development of LBr potato varieties adapted to local environments is thus a potentially very high reward, disruptive proposition. It is also a high risk proposition, facing numerous research and non-research related challenges, not least of which is a strong “anti-GM” lobby in many of the target populations.

The LBr potato project is structured in four consecutive stages. These stages and their associated costs and time durations are summarized in Table 1, based on figures documented by Schiek et al. [14]. This is a purely pedagogical exercise designed to illustrate the usefulness of the multistage ROV model derived in the previous sections. While the exercise is based on real project stage costs and time horizons, the other parameter values are hypothetical. The resulting calculations should therefore not be interpreted as an authoritative valuation of LBr potato research.

**Table 1:** Project to research and develop Late Blight resistant potato for release as a public good in two developing countries.

Stage	Cost (USD)	Duration	Time until completion	Description
1	531000	16	16	Basic replication and scaling up
2	336000	8	24	Multi-location/season testing
3	312000	4	28	Compilation of the regulatory dossier
4	181000	12	40	Deregulation
Launch	500000	–	–	Distribution/outscaling/ marketing of finished research product

Time expressed in annual quarters. Stage 1–4 costs and durations based on figures reported in [14].

The donor, USAID, originally awarded the LBr potato research contract to Cornell University in 2010, with planned release in India. However, stage 1 research and development at Cornell was unsuccessful. In 2015, USAID transferred the contract to Michigan State University (MSU), which proposed a different stage 1 research strategy, with planned release in Bangladesh and Indonesia instead of India. India was dropped as a target country partly because of the vigorous anti-GM lobby there, which made stage 4 success unlikely. MSU completed stage 1 successfully in 2019, and a couple years later USAID awarded them a new contract for stage 2 research and development.

In the multistage real options language developed above, USAID bought an option on stage 2 of a 4 stage LBr potato research project when it awarded the original contract to Cornell; but this option expired out of the money at the end of stage 1. USAID then again bought an option on stage 2 research when it transferred the contract to MSU. This time, the option expired in the money, triggering investment in stage 2 research. The investment in stage 2 research is an option on stage 3.

The aim of the present exercise is to calculate the ROV of stage 1 of the 4-stage LBr potato research project that USAID awarded to MSU in 2015. This is the value of USAID’s option to fund stage 2 once stage 1 is complete. The exercise is conducted from the donor’s perspective in the months prior to the “go/no-go” stage 1 investment decision. All values are given in terms of 2015 US dollars.

In order to make the calculation, the research stage and final launch costs $K_{0},K_{1},...,K_{4}$ and time horizons $T_{0},T_{1},...,T_{3}$ listed in Table 1 are required, as well as the quarterly discount rate *r*, the project NPV volatility *s*, and the project NPV at the time of the go/no-go decision to invest in stage 1 LBr research, *x*(0).

USAID generally uses a high annual discount rate of 0.12 in its cost-benefit analyses of agricultural development projects [56]. A high discount rate is reasonable considering the long time horizon and high uncertainty surrounding project NPV, and considering that any alternative use of USAID funds would entail a similar level of elevated risk. The same annual (discrete) discount rate is thus adopted in the present exercise, which works out to a (continuous) quarterly discount rate of 0.028 used in the calculation.

At the 2015 go/no-go decision point, no published ex-ante impact assessments of LBr potato in the target countries exist. For the pedagogical purposes of this exercise, I suppose that the donor conducts its own ex-ante impact assessment internally, finding that annual net benefits are likely to scale sublinearly with time by 0.6% for every 1% increase in time, reaching $110 thousand in year 3 after release. Mathematically, this may be expressed


$$\begin{array}{c} y(t)=y(\hat{t}){\left(\frac{t}{\hat{t}}\right)}^{\alpha } \end{array}$$ (48)

Where *y*(*t*) stands for LBr potato net benefit at *t* annual quarters after release, $y(\hat{t})=$ $110000, α= 0.6; and $\hat{t}=$ 12 quarters (i.e., 3 years).

**Fig 2:**
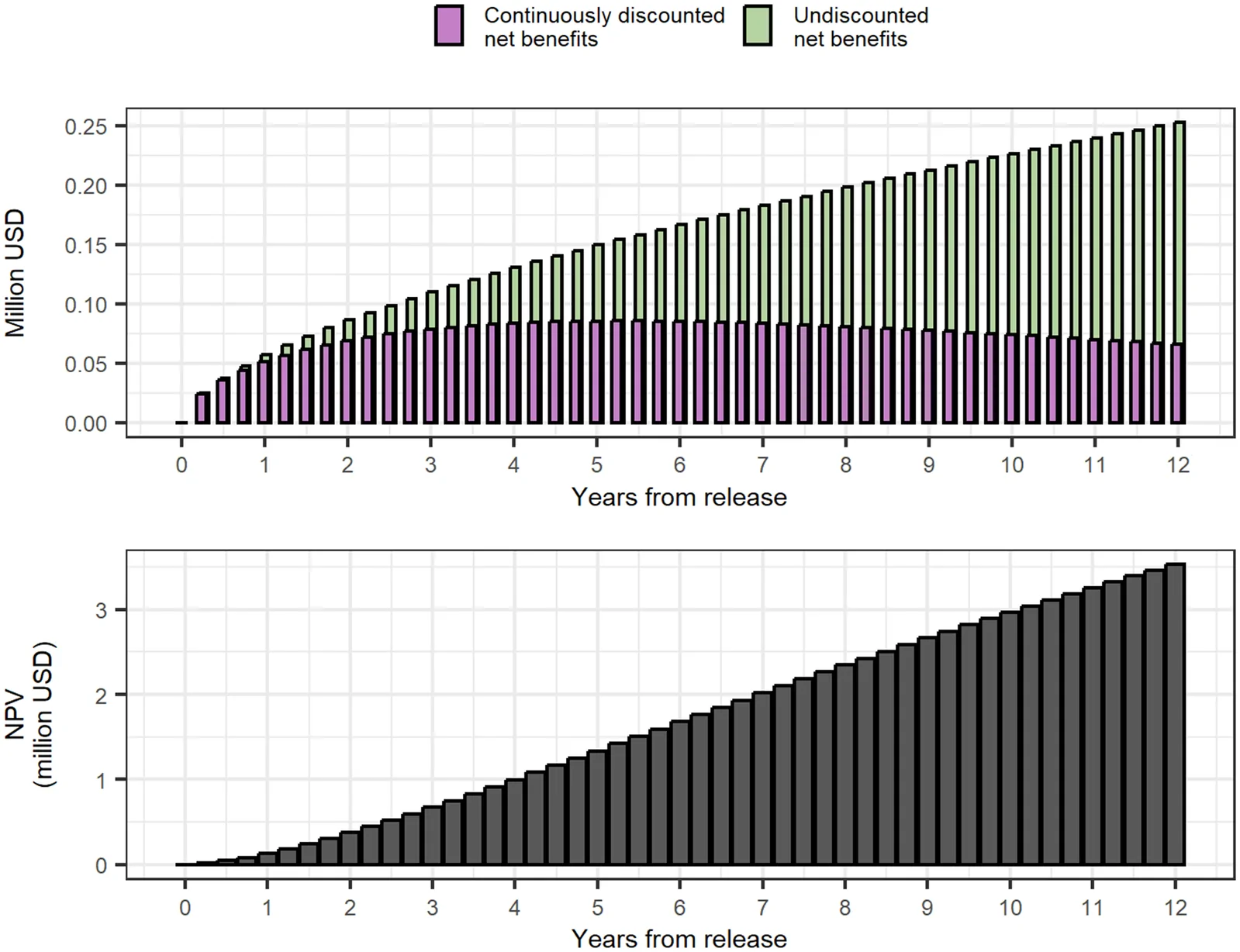
A hypothetical 12 year projection of the discounted and undiscounted LBr potato net benefit stream from time of release in the target countries. (Top) Projection from time of release in the target countries. (Bottom) The integral of discounted net benefits up to each year.

The corresponding continuously discounted net benefit stream (*e*^-*rt*^ *y*(*t*)) is overlaid onto the undiscounted stream (*y*(*t*)) in the top panel of Fig 2. (Again, this is a hypothetical projection for pedagogical purposes. A recent ex ante impact assessment suggests that net benefits could be substantially higher [57].) Integration of the discounted net benefits with respect to *t* over $\left[T_{0},T_{-1}\right]$ gives project NPV at time of release (*x*(*T*_0_)).


$$\begin{array}{cc} x(T_{0}) & =\int_{T_{0}}^{T_{-1}}e^{-rt}y(t)\;dt\\ & =\frac{y(\hat{t})}{{\hat{t}}^{\alpha }r^{\alpha +1}}(-\Gamma (\alpha +1,r(T_{-1}-T_{0}))+\Gamma (\alpha +1,0)) \end{array}$$ (49)

Where Γ(u,v) is the incomplete gamma function. The upper bound *T*_-1_ is a net benefit cutoff set by the donor. Net benefits continue beyond *T*_-1_, but the donor is only interested in net benefits up to *T*_-1_. Values of *x*(*T*_0_) are given in the bottom panel of Fig 2 for cutoffs ranging from 1 to 12 years after release. For the calculation of ROV in the present hypothetical exercise, the donor sets *T*_-1_–12 years (48 quarters) after release. This gives a project NPV of $3.459 million at time of release *T*_0_, i.e., at the end of stage 4 research. The project NPV discounted back to the time of the donor’s go/no-go decision to fund stage 1 research is then $x(0)=e^{-rT_{4}}x(T_{0})=$ $1.129 million.

The only remaining information required to calculate ROV is the LBr potato release and outscaling cost due at *t* = *T*_0_ upon successful completion of stage 4, and the project NPV volatility parameter *s*. For this pedagogical exercise, it is hypothesized that the donor estimates the release and outscaling cost to be $500 thousand. Because no project NPV time series exists whereby project volatility *s* might be directly estimated, an implicit value for *s* must be deduced. One practical way to do this is to calculate *s* based on expert elicitation of the upper and lower bounds on *x*(*T*_0_). That is to say, interpreting these elicited bounds as the 95% confidence interval, it is then straightforward to solve for *s* based on the assumption of lognormally distributed *x*(*T*_0_). In the present exercise, I hypothesize that such elicitation results in a value of *s* = 0.266. This corresponds to a coefficient of variation ($s/(r\sqrt{T_{0}})$) of 1.5, reflecting the high uncertainty that typically surrounds the expected impact of disruptive new technology a decade in advance of its completion and release.

With all of the necessary information in hand, the ROV of stage 1 LBr research is calculated by first calculating the ROV of stage 4, the last stage, and then calculating the ROV of stage 3, and so forth, recursively, as explained in the previous section. In the present exercise, this results in a stage 1 LBr research ROV of $613667, which exceeds the stage 1 cost of $531000 by $82667, resulting in a “go” investment decision. On the ROV basis, then, the stage 1 investment is justified. By the conventional CBA criterion, meanwhile, the project would be rejected, as the left-hand side of inequality (7) works out to $508913, which falls below the stage 1 cost by $22087.

The recursively calculated ROVs for each stage of research are presented in Table 2. Also included in this table are each stage’s ROV elasticity with respect to project NPV and probability of finishing in the money, which are calculated in the process. This provides additional information, not provided in conventional CBA, that the donor may find useful in their decision making process.

**Table 2:** LBr potato project stage 1-4 ROV.

Stage	ROV	ROV elasticity w.r.t. project NPV	Probability of success
1	613667	1.257	0.667
2	794250	1.169	0.674
3	926429	1.137	0.799
4	1000979	1.101	0.622

In cases where no ex-ante impact assessment of project NPV is available, it may be instructive to solve ROV of stage 2 research for the break even project NPV, i.e., the value of *x*(0) required to yield an ROV equal to the stage 1 cost. In this example, the break even project NPV is $1.035 million. Any project NPV above this value results in an ROV of stage 2 LBr research greater than the investment required to implement stage 1, and thus in a go decision.

## 5. Discussion and conclusion

The ROV method presented in this article is novel in two regards. First of all, it accommodates the multistage structure of AR4D projects. The key step in the derivation permitting this development is the demonstration via Itô’s lemma that if project NPV is lognormally distributed, then ROV itself is also lognormally distributed with computable parameters given in Eqs (38). Since the single stage ROV formula applies to any lognormally distributed variable, ROV itself may then be fed back into this formula to evaluate the ROV of ROV; and this may be repeated to evaluate the stage 1 ROV of a project of any number of stages. In the process of computing the stage 1 ROV of a multistage project, the ROV of all subsequent project stages, together with their respective probability of success and sensitivity to changes in the underlying project NPV, are also computed, providing the donor with potentially useful complementary information on which to base the strategic go/no-go funding decision for stage 1.

Secondly, whereas most ROV methods build off of the foundations of financial option pricing—often leading to strained attempts to construe projects as traded goods in efficient markets—here the method is built upon suitable non-market foundations. These non-market foundations are considerably simpler than their financial counterparts. Instead of the replicating portfolios/complete markets and no-arbitrage rule assumed in financial and corporate contexts—assumptions which themselves rest upon broader conditions regarding competitive market equilibrium and informational efficiency—the method presented here rests on two required conditions: 1) that NPV and ROV are expected to grow at the discount rate (something that is definitionally true and so does not need to be assumed); and 2) that future project NPV is approximately lognormally distributed through time—i.e., that the evolution of project NPV is reasonably well approximated by gBm.

To justify the latter condition, which is often contested in the ROV literature, I argue that the discontinuities practitioners seek to accommodate through jump or jump-diffusion models are often artifacts of reporting protocols rather than features of the value-generating process that is reported. In the AR4D context, the relevant object of analysis is not the discretely reported value trajectory, but the latent evolution of expected social value itself, which may plausibly be treated as continuous. I further address the related concern that project value may be path dependent, and hence not well approximated by a Markov process. While historical contingencies undoubtedly shape underlying technological and institutional dynamics, standard results in financial economics and valuation practice suggest that, under rational updating, current value may be treated as a sufficient summary of decision-relevant information for practical purposes. Together, these considerations clarify the conditions under which the latent project value process may be reasonably approximated as continuous and Markovian. Finally, even if substantial discontinuities or path-dependent effects arise over the full 10–20 year horizon of a typical AR4D project, the multistage framework substantially shortens the effective valuation horizon over which these approximations must hold, typically to sequential decision intervals on the order of 1–5 years.

The multistage ROV model derived in this article is not limited to transgenic projects of the type explored in the illustrative example above, but may be applied more broadly in any non-market research setting where the conditions just described are plausibly met. This includes conventional crop and livestock breeding projects, and also potentially extends to projects not necessarily focused on genetic gain, or that involve a considerable socio-ecological component—e.g., agroecology, climate-smart agriculture, value chain integration, and payments for ecosystem services—so long as the stated conditions remain defensible.

## Supporting information

S1 Appendix Mathematical proofs and derivations. Includes a proof of Eq 13 by straightforward integration, and a derivation of Eq 14 from the Black-Scholes PDE.
(PDF)
