# Supplementary material for: The real option value of multistage agricultural research for development
Source: PLoS One. 2026 Sep 17;21(9):e0336819. doi: 10.1371/journal.pone.0336819 (PMC13585265; doi:10.1371/journal.pone.0336819)
Supplement: Supplementary file 1 [file pone.0336819.s001.pdf]

S1 Appendix: Mathematical Proofs and Derivations

Proof of Eq 13 through straightforward integration

(The proof below closely follows that of Hull in the appendix to chapter 13 of his book [19].)

By definition, for a random variable q and a constant C,

$$E[\max(q-C,0)] = \int_{C}^{\infty} (q-C)p(q)\,dq \tag{A1}$$

Where $p(q)$ is the probability density function of the random variable q. If $\ln(q)$ is normally distributed with mean ν and variance ω^2, then

$$E[q] = e^{\nu+\frac{\omega^2}{2}} \tag{A2}$$

and

$$p(q) = \frac{1}{q\omega}\phi\left(\frac{\ln(q)-\nu}{\omega}\right) \tag{A3}$$

Where $\phi()$ is the standard normal probability density function.

Now, introducing a change of variables,

$$u = \frac{\ln(q)-\nu}{\omega} \tag{A4}$$

Such that

$$\frac{du}{dq} = \frac{1}{q\omega} \;\rightarrow\; dq = q\omega du \tag{A5}$$

The definition in Eq (A1) resolves as follows.

$$\begin{aligned}
E[\max(q-C,0)] &= \int_{\frac{\ln(C)-\nu}{\omega}}^{\infty} (e^{u\omega+\nu}-C)\phi(u)\,du \\
&= \int_{\frac{\ln(C)-\nu}{\omega}}^{\infty} e^{u\omega+\nu}\phi(u)\,du - C\int_{\frac{\ln(C)-\nu}{\omega}}^{\infty} \phi(u)\,du \\
&= \frac{1}{\sqrt{2\pi}}\int_{\frac{\ln(C)-\nu}{\omega}}^{\infty} e^{u\omega+\nu-\frac{u^2}{2}}\,du - C\Phi\left(-\frac{\ln(C)-\nu}{\omega}\right) \\
&= \frac{1}{\sqrt{2\pi}}\int_{\frac{\ln(C)-\nu}{\omega}}^{\infty} e^{-\frac{(u-\omega)^2}{2}+\nu+\frac{\omega^2}{2}}\,du - C\Phi\left(-\frac{\ln(C)-\nu}{\omega}\right) \\
&= e^{\nu+\frac{\omega^2}{2}}\frac{1}{\sqrt{2\pi}}\int_{\frac{\ln(C)-\nu}{\omega}}^{\infty} e^{-\frac{(u-\omega)^2}{2}}\,du - C\Phi\left(-\frac{\ln(C)-\nu}{\omega}\right) \\
&= E[q]\Phi\left(-\frac{\ln(C)-\nu}{\omega}+\omega\right) - C\Phi\left(-\frac{\ln(C)-\nu}{\omega}\right)
\end{aligned} \tag{A6}$$

Note that Eq (A2) can be rearranged into an expression for ν.

$$\nu = \ln(E[q]) - \omega^2/2 \tag{A7}$$

Substituting this for ν in the definition gives

$$E[\max(q-C,0)] = E[q]\Phi\left(\frac{\ln\left(\frac{E[q]}{C}\right)+\frac{\omega^2}{2}}{\omega}\right) - C\Phi\left(-\frac{\ln\left(\frac{E[q]}{C}\right)-\frac{\omega^2}{2}}{\omega}\right) \tag{A8}$$

■

Derivation of Eq 14 from the Black-Scholes PDE

Starting from the Black-Scholes PDE,

$$fr = \frac{\partial f}{\partial x}xr + \frac{\partial f}{\partial t} + \frac{s^2x^2}{2}\frac{\partial^2 f}{\partial x^2} \tag{A9}$$

Note that

$$x^2\frac{\partial^2 f}{\partial x^2} = \frac{\partial^2 f}{\partial \ln(x)^2} - \frac{\partial f}{\partial \ln(x)} \tag{A10}$$

Such that the PDE may be rewritten

$$fr = \frac{\partial f}{\partial \ln(x)}\left(r - \frac{s^2}{2}\right) + \frac{\partial f}{\partial t} + \frac{s^2}{2}\frac{\partial^2 f}{\partial \ln(x)^2} \tag{A11}$$

Defining $f = e^{-r\tau}y$, where $\tau = T - t$ for some future time T, and substituting this in for f,

$$0 = \frac{\partial y}{\partial \ln(x)}\left(r - \frac{s^2}{2}\right) + \frac{\partial y}{\partial t} + \frac{s^2}{2}\frac{\partial^2 y}{\partial \ln(x)^2} \tag{A12}$$

Substituting $T - \tau$ for t and expressing the equation in terms of a new function $v(u,\tau)$, where $u = \ln(x) + (r - s^2/2)\tau$,

$$0 = \frac{s^2}{2}\frac{\partial^2 v}{\partial u^2} - \frac{\partial v}{\partial t} \tag{A13}$$

This is the one dimensional heat equation, with fundamental solution

$$v(u,\tau) = \frac{1}{\sqrt{2\pi s^2\tau}}\int_{-\infty}^{\infty} v(\ell,0)e^{-\frac{(u-\ell)^2}{2s^2\tau}}\,d\ell \tag{A14}$$

The boundary condition of interest is $f(x,T) = \max(x(T) - K, 0)$, which in terms of $v(u,\tau)$ is $v(u,0) = \max(e^u - K, 0)$.

$$\begin{aligned} v(u,\tau) &= \frac{1}{s\sqrt{2\pi\tau}}\int_{\ln(K)}^{\infty}(e^{\ell} - K)e^{-\frac{(u-\ell)^2}{2s^2\tau}}\,d\ell \\ &= \frac{1}{s\sqrt{2\pi\tau}}\int_{\ln(K)}^{\infty} e^{\ell-\frac{(u-\ell)^2}{2s^2}}\,d\ell - \frac{K}{s\sqrt{2\pi\tau}}\int_{\ln(K)}^{\infty} e^{-\frac{(u-\ell)^2}{2s^2\tau}}\,d\ell \end{aligned} \tag{A15}$$

Introducing the substitution

$$z = \frac{\ell - u}{s\sqrt{\tau}} \rightarrow\ s\sqrt{\tau}dz = d\ell \tag{A16}$$

The integral simplifies to

$$\begin{aligned} v(u,\tau) &= \frac{1}{\sqrt{2\pi}}\int_{\frac{\ln(K)-u}{s\sqrt{\tau}}}^{\infty} e^{zs\sqrt{\tau}+u-\frac{z^2}{2}}\,dz - \frac{K}{\sqrt{2\pi}}\int_{\frac{\ln(K)-u}{s\sqrt{\tau}}}^{\infty} e^{-\frac{z^2}{2}}\,dz \\ &= \frac{1}{\sqrt{2\pi}}\int_{\frac{\ln(K)-u}{s\sqrt{\tau}}}^{\infty} e^{-\frac{(z-s\sqrt{\tau})^2}{2}+u+\frac{s^2\tau}{2}}\,dz - K\Phi\left(-\frac{\ln(K)-u}{s\sqrt{\tau}}\right) \\ &= e^{u+\frac{s^2}{2}\tau}\frac{1}{\sqrt{2\pi}}\int_{\frac{\ln(K)-u}{s\sqrt{\tau}}}^{\infty} e^{-\frac{(z-s\sqrt{\tau})^2}{2}}\,dz - K\Phi\left(-\frac{\ln(K)-u}{s\sqrt{\tau}}\right) \\ &= e^{u+\frac{s^2}{2}\tau}\Phi\left(\frac{-\ln(K)-u}{s\sqrt{\tau}} + s\sqrt{\tau}\right) - K\Phi\left(-\frac{\ln(K)-u}{s\sqrt{\tau}}\right) \end{aligned} \tag{A17}$$

Expressing everything once more in terms of $y(x,\tau)$, this reduces to

$$y(x,\tau) = e^{r\tau} x(t)\Phi(\delta + s\sqrt{\tau}) - K\Phi(\delta) \tag{A18}$$

Where

$$\delta = \frac{\ln\left(\frac{x(t)}{K}\right) + \left(r - \frac{s^2}{2}\right)\tau}{s\sqrt{\tau}} \tag{A19}$$

Multiplying through by the discount factor gives the expression for $f(x,\tau)$.

$$f(x,\tau) = x(t)\Phi(\delta + s\sqrt{\tau}) - e^{-r\tau} K\Phi(\delta) \tag{A20}$$

Which is equivalent to Eq 14 when $t = 0$, $T = T_0$, $K = K_0$.
■
